# Disrupted identities, invisibility and precarious support: a mixed methods study of LGBTQI adolescents and young adults with cancer

**DOI:** 10.1186/s12889-023-16739-9

**Published:** 2023-09-21

**Authors:** Jane M. Ussher, Kimberley Allison, Rosalie Power, Samantha Ryan, Janette Perz, The Out with Cancer Study Team, Alexandra Hawkey, Chloe Parton, Cristyn Davies, Lucy Watson, Fiona E. J. McDonald, Antoinette Anazodo, Martha Hickey, Kerry H. Robinson, Katherine Boydell, Jenni Bruce, Julie Rae, Tenley Gilmore

**Affiliations:** Translational Health Research Institute, School of Medicine, Western Sydney University, Sydney, Australia

**Keywords:** Cancer, Adolescent and Young Adult (AYA), LGBTQI, Mixed-method, Qualitative, Psycho-social, Disclosure, Identity, Social support, Health-care professionals

## Abstract

**Background:**

Lesbian, gay, bisexual, transgender, queer and intersex (LGBTQI) adolescents and young adults (AYAs) with cancer report higher levels of depression and anxiety and lower health related quality of life than non-LGBTQI AYAs with cancer, and LGBTQI adults with cancer. This mixed methods study examined LGBTQI AYAs' experiences of cancer and cancer care, to understand these health disparities.

**Methods:**

Online surveys were completed by 95 LGBTQI AYAs with cancer (age 16–39 years); 19 AYAs took part in a one-to-one semi structured interview. Reflexive thematic analysis of interviews and open-ended survey responses facilitated in-depth examination of subjective experiences; descriptive statistics performed on individual closed-ended survey items identified the percentage of AYAs reporting experiences identified in the qualitative analysis.

**Results:**

63% of AYAs reported high or very high distress on the K10. Three themes were identified in the qualitative analysis: 1) “Identities in flux”, included subthemes “Cancer disrupts developing identities, and involvement with LGBTQI communities”; “Internalized prejudice impacts identities”; and “Cancer facilitates identities and embodiment”. 2) “Invisibility in cancer care”, included subthemes “Navigating disclosure amongst cis-heteronormative assumptions”, “Discrimination and paternalistic cancer care” and “ Cis-heteronormativity within cancer information”. 3) “Precarious social support for LGBTQI AYAs with cancer”, included subthemes “ Social support during cancer is helpful for LGBTQI AYAs”, “LGBTQI AYAs navigate limited support”, and“ Finding cancer peer support networks is difficult for LGBTQI AYAs”.

**Conclusions:**

LGBTQI AYAs with cancer experience psychosocial vulnerabilities related to identity development, experiences of care, and social support networks. These factors likely contribute to their previously evidenced elevated risk of distress, relative to both non-LGBTQI AYAs and LGBTQI older adults. AYAs affected by cancer may require additional, tailored supportive care, including targeted information resources, LGBTQI AYA specific cancer support groups, or partnerships and referrals to LGBTQI community organisations. Additionally, it is evident that health care professionals and cancer services have much work to do in ensuring LGBTQI AYAs receive affirming and appropriate care across paediatric and adult clinical settings. They must move beyond assuming all patients are cisgender, heterosexual and do not have intersex variations unless otherwise stated; work to signal inclusivity and facilitate disclosure; and be able to respond appropriately with tailored information and care, which is inclusive of LGBTQI partners, chosen family, and support systems.

## Background

Lesbian, gay, bisexual, transgender, queer and intersex (LGBTQI) communities are increasingly recognised as a “growing and medically underserved” population in cancer care [1]. In comparison to the cisgender, heterosexual cancer population, LGBTQI adults with cancer report higher levels of distress, manifested by higher rates of depression and anxiety and lower health related quality of life [2–5], as well as lower satisfaction with care [6, 7]. Globally over a million adolescents and young adults (AYAs, age 15–39 years) are diagnosed with cancer each year [8], with 1,233,225 cases estimated in 2020 [9]. While research in the experiences and needs of LGBTQI AYAs with cancer is limited [10], there are indications that this group report worse psychological outcomes than non-LGBTQI AYAs with cancer. For example, it has been reported that sexuality diverse AYA women with cancer are more likely to be depressed than their heterosexual counterparts [11], and LGBTQI AYAs with cancer were more likely to experience anxiety and depression during the COVID-19 pandemic than non-LGBTQI counterparts [12]. Poorer psychosocial outcomes in LGBTQI AYAs with cancer are associated with unique challenges arising from the intersections between cancer experience, youth, and membership of a marginalised group. These include minority stressors common across LGBTQI age groups, such as discrimination [2, 13], poorer support from biological family, cancer peer groups and support services [12, 14, 15], financial hardship due to anti-LGBTQI hostility in the workplace [12], difficulties in having to repeatedly navigate disclosure of LGBTQI status [10, 16–18], invisibility of LGBTQI people in healthcare systems [17, 19], and unique impacts of cancer on LGBTQI identities and community connections [20–22].

However, LGBTQI AYAs with cancer experience several specific concerns. Adolescence and young adulthood are critical developmental stages, with AYAs often newly exploring, establishing and articulating sexuality and gender identities, relationships and communities [23–25], meaning that these aspects of life may be particularly vulnerable to disruption by cancer. AYAs’ relative lack of experience with healthcare systems and lack of health literacy [26] may result in additional challenges, including dealing with health care professional (HCP) assumptions that patients are heterosexual and cisgender (cis-heteronormativity), or anti-LGBTQI prejudices, and difficulties in finding supportive HCPs or appropriate information and services [10, 16, 18, 27]. LGBTQI cancer information resources focus on older adults [19], meaning that AYAs are an overlooked minority in LGBTQI cancer care. Greater clinical and research attention is necessary to ensure that LGBTQI AYAs can access culturally competent, safe and affirming cancer care. As much of the existing literature has focused on HCP and researcher perspectives on LGBTQI AYA cancer, e.g., [18, 27, 28], there is a need for person-centered research focused on the perspectives of AYAs [18, 27, 28].

The present analysis aimed to address this gap in the research literature by examining subjective experiences of cancer and cancer care among LGBTQI AYAs, drawing on the qualitative and survey findings from the mixed method Out with Cancer study [13, 19–21, 29, 30]. This complements quantitative analysis from this study, which found higher rates of distress and lower quality of life (QOL) in AYAs compared to older LGBTQI adults with cancer, associated with greater minority stress and discrimination, more impact of cancer on their gender identity and less social support in AYAs compared to older LGBTQI adults [13]. In addition, LGBTQI AYAs with cancer were less likely to have disclosed their sexuality or gender identity to friends, family and HCPs [13]; were less able to find LGBTQI-appropriate cancer information, and were less satisfied with care, compared to older LGBTQI adults [13, 29]. This paper enables further interpretation of these findings, through in-depth qualitative examination of AYA experiences, complemented by survey responses.

## Method

### Study design

A combination of online survey and one-to-one interviews was used to examine the subjective experiences of cancer and cancer care among LGBTQI AYAs. The interviews facilitated in-depth examination of the subjective interpretation, meanings and perceived consequences of AYA experiences; the survey provided information on the percentage of a broad sample of AYAs reporting the experiences identified in the qualitative analysis. The study was part of the broader Out with Cancer study [13, 19–21, 29, 30]. which explored LGBTQI cancer care from the perspectives of LGBTQI people with cancer (AYAs and older adults), carers and healthcare professionals through online surveys and semi-structured interviews. The project was underpinned by principles of integrated knowledge translation (iKT) [31], with a stakeholder advisory committee comprising LGBTQI people with cancer and carers, cancer HCPs, and representatives from cancer support and LGBTQI health organisations involved in co-design and co-production at all stages of the research. Ethics approval was provided by Western Sydney University Human Research Ethics Committee (H12664). All participants provided written informed consent. All methods were carried out in accordance with relevant guidelines and regulations outlined in the Declaration of Helsinki.

### Participants and recruitment

Participants were eligible for the broader Out with Cancer study if they: (a) identified as LGBTQI; (b) had been diagnosed with cancer or had undergone medical intervention related to cancer risk; and (c) were 16 years or older at the time of the study. In addition to broad recruitment strategies (social media advertisements, cancer and LGBTQI community organisations, in-person LGBTQI events), specific strategies to recruit LGBTQI AYAs with cancer included advertising via community organisations for young people impacted by cancer (Canteen Australia/Aotearoa, including their LGBTQI support group) and LGBTQI young people (Twenty10), as well as via healthcare professionals working in the Australian Youth Cancer Services. Snowball recruitment was also employed, with participants encouraged to share the survey link with other people who might be eligible. Surveys and interviews were completed between September 2019 and September 2021. This paper draws on data from the AYA subset of the broader sample, using an internationally established definition of AYA [32]. This included 95 surveys and 19 semi-structured interviews of participants aged 16–39 years at the time of data collection. Focus on current AYA status, rather than AYA status at time of diagnosis, was a consensus decision arrived at within the stakeholder group, as several scales focused on feelings at the present time, and the interviews focused on being an AYA cancer survivor.

### Survey and measures

The survey was administered online, to facilitate anonymity and ease of access through a link provided through online recruitment materials. The survey included a combination of validated measures, closed-ended demographic questions and open-ended prompts, described in detail elsewhere [13]. The ten-item Kessler Psychological Distress Scale (K10) [33] asked participants to rate the frequency of distressing feelings over the past four weeks, to produce a total distress score (range 10–50), which can then be categorized as low (10–15), moderate (16–21), high (22–29) or very high (30–50) distress [34]. The remaining items used in this analysis were derived from existing LGBTQ surveys, presented in Likert scales, and included: items on minority stress, asked using separate wording for LGBQ, trans and gender diverse (TGD) and intersex participants—discomfort being LGBTQI and control over disclosure [35–37], discrimination in general life and in cancer care [2], outness in general life and to HCPs [38]; physical concerns relating to cancer and treatment impacts on the body (e.g. hair loss, scarring) [4]; sexual concerns [39]; impacts on LGBTQI identity, based on the format of the Illness Intrusiveness Ratings Scale [40]; ability to find helpful information about being a LGBTQI person with cancer, derived from the Health Literacy Questionnaire (HLQ) [41]; and social support, using the five-item social support subscale of the Health Literacy Questionnaire [41]. After each measure a prompt asked participants, “Is there anything else you would like to tell us about this?”.

### Semi-structured interview procedure

Semi-structured interviews were conducted individually via phone or videoconferencing software and lasted approximately one hour. The interview schedule asked about participants’ experiences of cancer and cancer care, including their interactions with HCPs, their support networks, and how cancer had impacted their lives. Questions were tailored for each interview based on participants’ survey responses to probe their experiences in more detail.

### Analysis

#### Descriptive statistics

Frequency data and percentages were collected for responses to the closed survey items. Likert scale results were dichotomized to agree-disagree, to aid in readability and interpretation alongside the qualitative data, a practice adopted in previous mixed methods cancer research [22, 42]. The results of the total validated scales have been reported elsewhere [13].

### Qualitative analysis

Open-ended survey responses and interviews were analyzed using reflexive thematic analysis, to facilitate the identification and analysis of patterns or themes within the data [43]. The interviews were transcribed verbatim and checked for errors by listening to the interview recording whilst reading the transcript. During this process identifying information was removed from the transcripts, and participant names were replaced with pseudonyms. Through a collaborative process with our stakeholder advisory committee, a subset of interview transcripts was then read line by line, with notes made to capture first order codes and concepts such as “AYA-specific issues” and “impact of cancer on LGBTQI identity”. Through discussion and decision-making, the final coding frame was formulated which included codes such as “cancer as a barrier/ inhibitor to identities” and “culturally safe care, services, and support”. The interview data and open-ended survey responses were then imported into NVIVO and coded. When coding was complete, each code was summarized. This helped to identify commonalities within codes and across the data. The summaries were then read, commented on by all authors, and re-organized into preliminary themes. All authors read and commented on the preliminary themes, and through a discussion process, the themes were re-organized and refined. Members of the stakeholder advisory committee then read and provided comment on the interpretation and reporting of the data. The themes were further refined, including incorporating feedback on language and interpretation. In addition to team analysis, strategies to address research rigour included prolonged engagement with the subject matter, a detailed audit trail, and reflexivity. In the presentation of results, pseudonyms are used for quotes from interviewees, while survey quotes are identified as “survey”; demographic details of age, sexual and gender identity and/or intersex traits and cancer type are provided.

## Results

### Participant characteristics

The demographic and cancer characteristics of survey and interview participants are presented in Tables 1 and 2. Most participants were in the young adult age group (mean age 29.4 surveys, 32.4 interviews), lived in Australia (62.1% surveys, 78.9% interviews) and were Caucasian (71.3% surveys, 76.5% interviews). Considerable diversity was present in participants’ gender and sexual identities, regionality (Table 1) and cancer diagnoses (Table 2).

**Table 1:** Demographics of survey and interview participants

**Demographic Characteristic**	**Survey participants** (*n* = 95)	**Interview participants** (*n* = 19)
	*M (SD),* range	*M (SD),* range
Age at time of study (years)	29.4 (6.7), 16–39	32.4 (5.1), 21–39
Age at diagnosis (years)	24.3 (8.5), 1–36	28.8 (5.4), 16–36
	*n* (%)	*n* (%)
Country		
Australia	59 (62.1%)	15 (78.9%)
United States of America	21 (22.1%)	2 (10.5%)
United Kingdom	4 (4.2%)	1 (5.3%)
New Zealand	2 (2.1%)	0 (0.0%)
Canada	2 (2.1%)	0 (0.0%)
Other^a^	7 (7.4%)	1 (5.3%)
Location		
Urban	48 (51.1%)	7 (41.1%)
Regional	40 (42.6%)	9 (52.9%)
Rural or remote	6 (6.4%)	1 (5.9%)
Race/ethnicity		
Caucasian	67 (71.3%)	13 (76.5%)
Asian	5 (5.3%)	1 (5.9%)
Australian Aboriginal, Torres Strait Islander or Māori	2 (2.1%)	0 (0.0%)
Mixed background	10 (10.6%)	2 (11.8%)
Other/unclear background^b^	10 (10.6%)	1 (5.9%)
Gender		
Cis female	42 (44.2%)	6 (31.6%)
Cis male	22 (23.2%)	5 (26.3%)
Trans female	3 (3.2%)	1 (5.3%)
Trans male	2 (2.1%)	0 (0.0%)
Non-binary	21 (22.1%)	6 (31.6%)
Different or multiple identities	5 (5.3%)	1 (5.3%)
Sexuality		
Lesbian, gay or homosexual	44 (46.3%)	11 (57.9%)
Bisexual or pansexual	22 (23.2%)	3 (15.8%)
Queer	21 (22.1%)	4 (21.1%)
Straight or heterosexual	4 (4.2%)	0 (0.0%)
Different or multiple identities^c^	4 (4.2%)	1 (5.3%)
Intersex variation		
Yes	13 (13.7%)	0 (0.0%)
No	75 (78.9%)	18 (94.7%)
Prefer not to answer	7 (7.4%)	1 (5.3%)
Relationship status^*^		-
Not in a relationship	28 (35.9%)	
Casually dating	6 (7.7%)	
Relationship with one other person	39 (50.0%)	
Multiple relationships	8 (10.3%)	
Education		
Less than secondary	6 (6.3%)	0 (0.0%)
Secondary	9 (9.5%)	0 (0.0%)
Some post-secondary	26 (27.4%)	3 (17.6%)
Post-secondary	54 (56.8%)	14 (82.4%)
Employment^*^		
Paid work	56 (58.9%)	10 (58.8%)
Unpaid work	14 (14.7%)	3 (17.6%)
Not engaged in work	27 (28.4%)	5 (29.4%)

^a^Surveys: Austria (*n* = 2), Chad, Costa Rica, Morrocco, Poland, Russia (*n* = 1 each). Interviews: Austria (*n* = 1)

^b^Surveys: Hispanic/Latinx (*n* = 3), Arabic, Jewish, Romani (*n* = 1 each), unclear (*n* = 4). Interviews: unclear (*n* = 1)

^c^Surveys: Lesbian/queer (*n* = 2), bicurious, different identity (*n* = 1 each). Interviews: lesbian/queer (*n* = 1)

*Participants could select multiple options

**Table 2:** Cancer characteristics of survey and interview participants

Cancer characteristic	Survey participants	Interview participants
	*n* (%)	*n* (%)
Cancer experience		
Had medical intervention for cancer risk	26 (27.4%)	2 (10.5%)
Diagnosed with cancer	73 (76.8%)	19 (100%)
Cancer diagnosis (first)		
Brain	5 (6.8%)	0 (0.0%)
Breast	9 (12.3%)	1 (5.3%)
Cervical	4 (5.5%)	0 (0.0%)
Colorectal	5 (6.8%)	1 (5.3%)
Leukaemia	8 (11.0%)	2 (10.5%)
Lymphoma	10 (13.7%)	3 (15.8%)
Ovarian	4 (5.5%)	0 (0.0%)
Sarcoma	4 (5.5%)	0 (0.0%)
Skin	7 (9.6%)	2 (10.5%)
Uterine	5 (6.8%)	4 (21.1%)
Other^a^	11 (15.1%)	5 (26.3%)
Not sure or unknown	1 (1.4%)	2 (10.5%)
Cancer stage		
Localised	40 (54.8%)	7 (41.2%)
Regional	16 (21.9%)	4 (23.5%)
Distant/metastatic	9 (12.3%)	2 (11.8%)
N/A	2 (2.7%)	1 (5.9%)
Not sure or unclear	6 (8.2%)	3 (17.6%)
Subsequent cancers^b^		
Recurrence	18 (24.7%)	4 (23.5%)
New primary cancer	4 (5.5%)	2 (11.8%)
Treatment status		
No treatment yet	3 (4.1%)	0 (0.0%)
On active curative treatment	6 (8.2%)	2 (11.8%)
On maintenance treatment	17 (23.3%)	7 (41.2%)
In remission/completed treatment	41 (56.2%)	5 (29.4%)
Receiving palliative care (no further active treatment)	1 (1.4%)	0 (0.0%)
Not sure, unclear, or multiple	5 (6.8%)	3 (17.6%)
Other chronic illnesses, disabilities, and impairments	27 (38.0%)	9 (52.9%)

^a^Surveys: head/neck, kidney, lung, testicular (*n* = 2 each), bladder, prostate, multiple (*n* = 1 each). Interviews: leukaemia, testicular (*n* = 2 each), bladder (*n* = 1)

^b^Participants could select multiple responses

Of the 68 AYAs who completed the K10, 12 (17.6%) reported low distress; 13 (19.1%) reported moderate distress; 17 (25.0%) reported high distress; and 26 (38.2%) reported very high distress.

The quantitative and qualitative findings related to minority stress, impact of cancer, experiences of cancer care and social support are integrated below, with the themes and sub-themes summarised in Table 3.

**Table 3:** Themes and subthemes identified from qualitative data

Themes	Subthemes
1. Identities in flux	1.1 Cancer disrupts developing identities, and involvement with LGBTQI communities 1.2 Internalized prejudice impacts identities 1.3 Cancer facilitates identities and embodiment
2. Invisibility within cancer care	2.1 Navigating disclosure amongst cis-heteronormative assumptions 2.2 Discrimination and paternalistic cancer care 2.2 Cis-heteronormativity within cancer information
3. Precarious social support for LGBTQI AYAs with cancer	3.1 Social support during cancer is helpful for LGBTQI AYAs 3.2: LGBTQI AYAs navigate limited support 3.3 Finding cancer peer support networks is difficult for LGBTQI AYAs

### Identities in flux

#### Cancer disrupts developing identities and involvement with LGBTQI communities

For many participants, cancer diagnosis and treatment were reported to have delayed or disrupted establishment of LGBTQI identities, impacting upon relationships and connections with LGBTQI communities and peers. Most participants agreed with the survey item “cancer impacted how I feel about being LGBTQI” (*n* = 52, 57.8%). Disruption of identity was more common for those who were newly exploring these aspects of themselves. Some participants reported that they were “still trying to figure everything out” and that “LGBTQI issues” had to be “put on the backburner”, due to having to “acutely survive this [cancer]” (Carter, 20, cis man, gay, leukaemia). Cancer was positioned as having “messed up” exploration of queer sexual relationships, a time when Brianna “didn’t know for sure” who she was (26, cis woman, queer, lymphoma). Others described establishing their identities post-cancer, telling us “when I was first diagnosed with cancer, I was didn't know I was non-binary” (Alex, 35, non-binary, gay, testicular) and “I've had strong feelings of regret that I didn't live as myself sooner and medically transition sooner” (survey, 32, non-binary, pansexual, testicular).

Persisting cancer-related ill health and reduced mobility prevented many participants from engaging in the socialization necessary to build relationships in LGBTQI communities, disrupting relational identities and belonging. Almost all AYAs reported physical (*n* = 65, 97.0%) and/or sexual (*n* = 43, 71.7%) concerns since cancer. Participants positioned these concerns as disqualifying or prohibiting them from engaging with young queer communities “based around nightlife” (Dylan, 32, non-binary, gay, leukaemia) and perceived to center around “going out clubbing, drinking or having sex” (Aaron, 32, cis man, gay, colorectal). Body image and sexual concerns led some cis gay men to feel “at odds with” the gay community (survey, 32, cis man, gay, colorectal) because it was “image conscious”, with resentment expressed towards the perceived “commercialist” (Oscar, 27, cis man, gay, lymphoma) and “transient” (Dylan, 32, non-binary, gay, leukaemia) nature of relationships. The physical inaccessibility of gay community spaces contributed to “struggles to find yourself in the gay community” (Aaron, 32, cis man, gay, colorectal). One survey respondent wrote,

I feel that my ability to explore my sexuality has been so severely impacted by my cancer treatment […] So much of queer sexuality and identity formation happens in nightclubs, bars, parties, events with alcohol and drugs – all spaces that I find difficult or impossible to access (survey, 26, cis woman, queer, sarcoma).

A survey respondent told us “pain post-surgery is horrendous, even 8 years later. I can't go out and be a part of the community. Let's face it, the queer community isn't overly disability friendly” (survey, 38, cis woman, lesbian, cervical). In this vein, over half of the participants agreed with the survey item “cancer impacted my involvement with LGBTQI communities” (*n* = 59, 65.6%).

Post-cancer changes in gendered embodiment, feelings of sexual attraction and sexuality also impacted upon LGBTQI identities. For example, one participant described reductions in her sexual desire as making her feel “more ‘straight’” and “extra asexual” (survey, 26, cis woman, queer, lymphoma), while another AYA woman who said “sex is painful and unpleasant” following cancer treatment, described having to “readjust what being queer means to me if I can’t have sex with women” (survey, 37, cis woman, queer, cervical). Some transgender AYAs noted that cancer treatment could have consequences for embodied gender – including “having a completely different body part to have body dysmorphia about” (survey, 37, non-binary and gender fluid, queer, BRCA) after removal of reproductive organs.

Having a radical cystectomy and removing parts of the female reproductive system has probably been challenging my feelings towards being more non-binary, which is a whole other confusing case. So that's an area that I'm still coming to terms with (Luca, 33, non-binary, queer, bladder).

The consequence for many AYAs was that cancer diagnosis and treatment could leave them worried about having to “catch up to other [LGBTQI] people my age” (survey, 28, cis man, gay, sarcoma).

#### Internalized prejudice impacts identities

AYAs still coming to terms with being LGBTQI may struggle with internalized prejudice. While the majority of AYA survey respondents agreed with the item “I am comfortable being LGBTQI” (*n* = 73, 80.2%), twelve (13.3%) agreed with the item “I wish I was not LGBTQI”. This was linked to broader issues of LGBTQI invisibility and societal discrimination and rejection, particularly for those who grew up in times or places where there was little LGBTQI representation or “where [trans] terms didn’t exist” (Dylan, 32, non-binary, gay, leukaemia). Almost all respondents (*n* = 82, 91.1%) had experienced discrimination in their lives. Dylan described how family prejudice and internalized “gay shame” meant they “grew up really distant from the queer community, and almost scared to be involved in it”, which continued into their early treatment experiences. A survey respondent felt that their “othering” from peers after their cancer diagnosis had caused them to reject their LGBTQI identity, as they “couldn't bear to have something else that made me different again” (survey, 25, cis woman, queer, sarcoma). Many AYAs were cautious about disclosing LGBTQI status to family and peers. For example, Luca (33, non-binary, queer, bladder) reported that they were “still figuring it out for myself, that’s why I’m not necessarily discussing it with others yet”. A non-binary intersex survey participant told us “there is so much internalized shame tied into the experience of being intersex, it's hard to disclose” (survey, 23, non-binary, bisexual, intersex, medical intervention). This was reflected in survey responses: while all respondents were out to at least some people in their general life, almost half (n = 38, 42.7%) agreed with the survey item “I keep careful control over who knows I am LGBTQI” and only thirteen (15.7%) were out to all family, friends and peers.

#### Cancer facilitates LGBTQI identities and embodiment

It is important to note that cancer and its treatment were not universally disruptive to LGBTQI identities and relationships. Three-quarters of respondents (*n* = 67, 74.4%) agreed with the survey item “cancer impacted how open I am about being LGBTQI”. AYAs described having “more courage” (Cara, 29, cis woman, gay, melanoma) to introduce partners to friends and family, “embrac[ing] the queer side [of myself]” (Dylan, 32, non-binary, gay, leukaemia) as a form of authentic self-expression and to promote LGBTQI inclusivity. As one survey respondent wrote, “having this cancer made me realise I will never be put in the closet again” (survey, 29, trans woman, pansexual, brain). Sexual intimacy could also be improved after cancer. Removal of breasts was described by a non-binary intersex participant as “a gamechanger when it comes to sex. Dysphoria no longer clouds the room like it used to” (survey, 22, genderqueer man, gay, intersex, medical intervention). Others reported increased ownership of their “kink” desires.

I’m a lot more sexually open now than what I was before. You know, like all my hard kinks and stuff I never really owned before having cancer. I sort of felt like I was a bit of a freak, never embraced it. But once I met [partner’s name] post cancer, [I thought] life is short, own it, do what you want. Do what makes you happy (Jake, 30, cis man, gay, testicular).

At the same time, “queer communities” were described as “more accepting” of cancer-related changes because of “understand[ing] that bodies are diverse” (survey, 34, cis women, queer, breast).

As a disabled woman, the effects of my cancer treatment also made me feel like it was impossible to achieve normative societal standards of womanhood. My scars and physical deformities and limb difference from cancer made me an "ugly" woman, or that I could never achieve a standard of womanhood that was expected of me. In some ways, discovering queerness alleviated this because I felt less pressure within queer spaces for my body to look a certain way, that within a queer identity there was more space for my disabled body to be accepted (survey, 25, cis-woman, queer, sarcoma).

In combination, these accounts in these three subthemes demonstrate the impact of cancer on the development of LGBTQI identities and connection with queer communities, at a time when AYAs were exploring what it means to be gay, lesbian, bisexual, queer or trans. For a minority, internalized prejudice associated with family prejudice and societal discrimination served as a compounding factor, adding to the negative impact of cancer on exploration and expression of sexuality and gender diversity. However, for others, cancer served to facilitate the open expression of LGBTQI identities and connection with queer communities.

### Invisibility within cancer care

The way in which HCPs responded to disclosure and open expression of LGBTQI identities had an impact on the wellbeing of AYAs. Navigating disclosure in the context of HCPs making cis-heteronormative assumptions was difficult. Many participants reported discrimination on the part of HCPs, or experienced paternalistic care. Cancer information is focused on cisgender, heterosexual, endosex populations, with little mention of LGBTQI people. In combination, this resulted in a feeling of invisibility in cancer care.

#### Navigating disclosure amongst cis-heteronormative assumptions

LGBTQI AYAs faced unique difficulties disclosing their diverse genders, sexualities and intersex variations within cancer care. Most participants kept careful control over the disclosure of their identities: only five (6.2%) participants were out to all their cancer HCPs; the majority (*n* = 62, 76.5%) disclosed selectively to only some HCPs, while 14 (17.3%) were not out to any of the HCPS involved in their cancer care. Participants explained that HCPs rarely asked about their diverse genders and sexualities and assumed they were straight and cisgender. Cara (29, cis woman, gay, melanoma) explained that one of their HCPs “just assumed straight away that my partner was my friend, and it’s awkward to correct him. That’s happened on a few occasions… it’s awkward and uncomfortable. And I think it probably makes my partner feel a little bit.. on the outskirts too”. Cis-heteronormative assumptions from HCPs made participants feel “alienated”, “awkward”, “silenced” and “pissed off”, forcing the “work” of disclosure upon participants: “heterosexuality remains the norm and the default. It forces us to do the work of coming out” (survey, 38, cis woman, queer, medical intervention).

However, challenging cis-heteronormative assumptions from HCPs was difficult for many participants. A survey participant (33, non-binary, queer, bladder) explained that they were “not fully out [to HCPs]” as “it is a newer self-process and not discussed with everyone”. Another participant said they were worried that if they came out to their HCP, this would then be shared with their parents: “The reason I haven’t [disclosed] is because I’m afraid they’ll say something to my parents by accident” (survey, 26, cis woman, queer, lymphoma). Disclosure takes “emotional energy” and is associated with fear of negative reactions.

Being gay, even now I'm twenty- there's always still that little bit of fear when you come out to people, even if it is in a sort of blasé, casual way. There's always that little bit inside you going, “Ooh.. what are they going to think?”, “how do they feel?”- which will probably be a forever thing, you never know how everybody individually is going to react (Carter, 20, cis man, gay, leukaemia).

This draining of emotional energy is greater when dealing with the symptoms of cancer, accompanied by fear of potential hostility from HCPs: “It isn’t safe [to disclose]. I don’t like having to justify myself ad infinitum” (survey, 35, non-binary, bisexual, breast); “sometimes I’m in too much pain or just too tired to deal with their unpredictable responses” (survey, 37, queer femme, queer, medical intervention); “I think there is a direct and depressing relationship between my loss of physical strength and the amount of emotional energy I’ve expended being scared of poor treatment and/or advocating or educating” (survey, 37, queer femme, queer, medical intervention). Some participants held a fear that disclosure would impact on their treatment and made efforts to avoid looking “alternative”: “I was very scared about my treatment if I told anyone. I already look alternative and even had a normal hair cut when I knew I had surgeries coming up” (survey, 37, non-binary, queer, medical intervention); “I don’t want them to know I’m gay because I don’t want them to treat me different” (Oscar, 27, cis man, gay, lymphoma). AYAs who were older reflected that fear of HCP negative reactions to disclosure were greater at a young age:

I'm sort of not comfortable until I can sort of gauge how I think they're going to respond. When I was sort of younger, I would care more how they would personally react. But as I’m getting older, I just care less about that (Aaron, 32, cis man, gay, bowel).

Non-disclosure of sexual and gender identities had negative consequences. It meant that participants were unable to bring their “full self” to their cancer care: “it feels sometimes you are complicating it by bringing your full self to the table” (Dylan, 32, non-binary, gay, leukaemia).

Non-disclosure also meant AYAs found it difficult to ask questions about their cancer diagnosis and the impact of their treatment, reporting that they were “too scared to ask” or “didn’t want to sound silly” (Cara, 29, cis woman, gay, melanoma), potentially missing out on important information for their cancer care.

I feel alienated when discussions around fertility and sexual health post cancer are so cis-heteronormative. I don't feel comfortable "outing" myself to my doctors in these contexts when it's assumed that fertility is related to heterosexual penetrative sex. I'm too scared to ask whether I'm able to preserve my fertility (eg freezing eggs? I don't even know what the options are because it's never been spoken about) so that a same-sex partner could carry a pregnancy, or any other alternative for queer pregnancies (survey, 26, cis woman, bisexual, lymphoma).

Not disclosing one’s identity was associated with participants feeling “fake” and like they were “lying”: “I feel like a bad human because I’m going against my value system of telling the truth, but I have to do that in order to preserve my mental health” (Jessie, 37, non-binary and genderfluid, queer, BRCA mutation). Non-disclosure meant that participants would not receive holistic cancer care.

Understanding a person's sexual identity preference, you need to be looking at it holistically, because you're not just looking at the anatomy anymore. You're looking at what that might mean to that person and what values and ideals they have behind it, which will change the treatment plan. It'll change the treatment process, which I realized when I was chatting with my specialist that we weren't coming from the same place and background. And that's why he wasn't fully understanding (Luca, 33, non-binary, queer, bladder).

There was a call for increased visibility and safety of LGBTQI AYAs in cancer care, as a survey participant (38, cis woman, queer, medical intervention) told us, “We are not in general afraid to come out/tick a box because it’s a “private matter”. We’re afraid health professionals won’t react well. WE WANT TO BE COUNTED. We want to be seen” (*capital letters in survey response*).

#### Discrimination and paternalistic cancer care

Fear of HCP hostility was a reality for many participants, with a substantial proportion (*n* = 41, 45.6%) reporting experiencing discrimination as part of their cancer care. As a survey participant (37, non-binary, lesbian and queer, unknown cancer) told us “I’ve been discriminated against by every health professional, other than those who are queer themselves. As an LGBTIQ + person in cancer care, choosing health professionals is about seeking the best worse option”. This discrimination included inappropriate comments, exclusion of partners, objectification, sexual harassment and paternalistic treatment. For example, a survey participant (39, cis woman, lesbian, cervical) reported “a doctor told me I shouldn’t have an issue with her putting her fingers inside of me “to test” something (no idea what to this day) because “people like you like this kind of thing”. Jessie (37, non-binary and genderfluid, queer, BRCA mutation) told us about the exclusion of their partner from an appointment with a medical professional:

I took her to the appointment, and he said it wasn’t protocol to have your partner in the room, but I’ve seen other people with their partners in those rooms. He just obviously didn’t like it. She just walked in, but he didn’t give her any eye contact. He didn’t acknowledge her.

In a similar vein, Ellen told us “It was horrible because he [HCP] didn't even acknowledge my partner in the room and treated us like we were little girls. So, we did not go back to that guy” (36, cis woman, lesbian, uterine). It was more common for trans participants to report experiencing transphobic discrimination during their cancer care (*n* = 13, 72.2%) than for LGBQ participants to report experiencing homophobic or biphobic discrimination (*n* = 31, 41.3%). For example, survey participants told us, “In group health sessions with allied health, or in hospital wards, there is so much transphobic/cis-normative talk between health workers and staff, I overhear it between them and it feels horrible” (survey, 37, non-binary and genderfluid woman, lesbian and queer, unknown cancer); “I don’t disclose that I’m intersex to everyone. Those who do know have not always responded positively unfortunately” (survey, 22, non-binary, bisexual, intersex, medical intervention).

Paternalistic treatment was evident in reports of feeling unheard by medical professionals and excluded from decision-making about their care, due to their young age and sexual identity. For example, Carter (20, cis man, gay, leukemia) reported: “Fertility preservation options were an issue with my treatment, as I had my reasons for not wanting preservation linked to my sexuality, that my healthcare team didn't get at best, or rejected completely as a regretful action at worst”. Conversely, Jade reported that her oncologist was “strict” and “wasn't very gay friendly”, as she would not approve saving Jade’s “eggs on ice” before surgery, to facilitate Jade having a “surrogate pregnancy later”, so Jade “left that hospital because of that” (Jade, 34, cis woman, lesbian, breast). A non-binary survey participant with breast cancer reported that medical professionals “did not want to listen” to them when they asked for a mastectomy, voicing that “I’d rather have them removed, they aren’t really important to me”, to which they were told “women at your age like to preserve their femininity however they can” (36, non-binary, bisexual, breast). As a result, this participant described that.

Every day I plan my next surgery and fear recurrence. And I resent the team of female doctors scrambling to save my poor 31-year-old breasts. And now I’m mutilated anyway. It’s never been safe, I don’t feel like it’s ever going to be safe. Why couldn’t they just listen to me.

Objectification of young trans and intersex bodies was commonly reported: “They simply don’t treat me as a person. I become an object and an issue as a trans person but also a young adult patient with cancer" (survey, 21, trans man, queer, melanoma).

The medical community has been nothing but abusive and exploitative regarding my intersex body. I've been subjected to medical photography, forced sedation, forced invasive examinations, forced surgical procedures, and lied to about needing surgical procedures under the claim that I had cancerous growths (33, non-binary, queer, intersex, medical intervention).

Because of experiencing discrimination and feeling excluded from their own care, LGBTQI AYAs reported feeling that their sexual and gender identities were “unwelcome” and invalidated in their cancer care. Dylan (32, non-binary, gay, leukemia) told us, “the environments are incredibly heteronormative and hetero type, so you instantly feel unwelcome and that permeates into every conversation you have from then on because you wonder what validity your identity has in that space”. For a survey participant, this negatively impacted their own feelings about their sexual identity: “cancer has never impacted on my ability to be open about my sexuality, it is the discrimination I have experienced from health professionals during my cancer care that has reduced my ability to be proud of who I am” (37, non-binary and gender fluid woman, lesbian and queer, unknown cancer type).

Several participants also commented on the importance of HCPs understanding the intersection of identities. For some, “cultural identity comes before my queer identity” (Jessie, 37, non-binary and genderfluid, queer, BRCA mutation). Others talked about the intersection of disability and queer identities “I would like extra support with being trans and autistic not just it being ignored so I can be treated like a cis male” (survey, 33, trans man, queer, uterine); “being a trans person with ASD and having cancer is really tricky because I have extra needs….I find that it’s really hard to have all those identities addressed and often one of them or all of them aren't addressed most of the time” (Howard, 33, trans man, queer, uterine). Finding “a LGBTQIA + knowledgeable and affirming practitioner who is accessible” in a rural or regional area was also problematic:

Like the other aspects of my identity it's very difficult to find an LGBTQIA+ knowledgeable and affirming practitioner who is accessible. If you live in a capital city you will find one….. In terms of being intersex, I wouldn't feel comfortable having a Pap smear in a regional rural area because my external genitalia look different. I have one cervix but two uteruses in the one space. It's very weird to bring it up especially when you consider my gender identity and the way that I look. I think most health professionals who didn't understand LGBTQIA+ issues intimately would be very confused, and this would impact on my care (survey, 22, genderqueer man, gay, intersex, medical intervention).

The consequence was that LGBTQI AYAs were often “referred to services in cities”, or experienced unmet needs in relation to their care.

#### Cis-heteronormativity within cancer information

LGBTQI AYAs were rendered invisible within cancer information resources for patients. Resources about cancer and being LGBTQI were reported as being “pretty much non-existent” (Aaron, 32, cis man, gay, bowel), with only 16 (19.8%) participants agreeing with the item “I am able to find helpful information about being a LGBTQI person with cancer”. For example, Aaron (32, cis man, gay, bowel) said that he “couldn’t find anything that’s specifically related to gay men going through it” and Dylan (32, non-binary, gay, leukaemia) reported that “all of the information out there, it’s all straight people on the leaflets and is about straight life”. Participants explained that there was a particular lack of information about sexual wellbeing for LGBTQI people with cancer, describing that there is “little consideration on how cancer care impacts the sex life of sexual minorities and how it is considered disposable in the face of survival conversations” (survey, 32, non-binary, gay, leukaemia). Information about sexual wellbeing after cancer was described as “incredibly heteronormative” (Dylan, 32, non-binary, gay, leukaemia) and “very generalized” (Carter, 20, cis man, gay, leukaemia), with “nothing at all about being in a female-female relationship (Cara, 29, cis woman, gay, melanoma).The same was said for information about fertility preservation procedures.

That whole [fertility preservation] process was incredibly heteronormative, the face to face of interactions were fine, but all the forms that I had to fill in well were heavily weighted not only towards heterosexual relationships, but also fairly conventional understandings of heterosexual relationships. So it was, “as a woman is your husband party to this process”, “as a husband, is your wife party to this process” (Joseph, 37, cis man, gay testicular).

Carter (20, cis man, gay, leukaemia) wished they had access to appropriate information around sexuality, reporting: “it would have been nice to have proper education around homosexual sex … because I’m missing out on a lot of school, I also missed the sex ed classes”.

In combination, the accounts in these three subthemes demonstrate the difficulties LGBTQI AYAs experience in navigating cis-heteronormative cancer care, including difficulties in disclosing identities to HCPs, the negative consequences of non-disclosure, feelings of invisibility in cancer information and support services, and the impact of discrimination or paternalistic care from HCPs.

### Precarious social support for LGBTQI AYAs with cancer

Social support is a protective factor for LGBTQI AYAs who are navigating cancer treatment and the impact of treatment on their identities. Most participants had strong support networks from family, partners and friends. However, a minority experienced vulnerability due to social isolation and the impact of anti-LGBTQI prejudice, as well as difficulties in calling on support from LGBTQI communities, not having an intimate partner to provide support, and the absence of cancer peer support networks for young queer people.

#### Social support during cancer is helpful for LGBTQI AYAs

For LGBTQI AYAs, support people played a unique and crucial role in navigation of cancer care. Most participants agreed that “I had strong support from family and friends” (*n* = 55, 57.9%), “could get access to several people who understand and support me” (*n* = 52, 54.7%) and “had at least one person who could attend medical appointments with me” (*n* = 60, 63.2%). When asked who their support people were during cancer, participants nominated their parents (*n* = 43, 55.1%), partners (*n* = 33, 42.3%), other family (*n* = 28, 35.9%) and friends (*n* = 30, 38.4%).

For example, Howard, a 33-year-old queer trans man with uterine cancer, said his dad was “very accepting and supportive” and helped him to attend cancer care at a women’s hospital. He said, “I'm glad my dad came with me because it wasn't as scary. I was in a room and there was like five other women, having my dad made me feel not as bad.” Participants who had partners also said their partners were “very emotionally and practically supportive” and helped them “advocate for myself and keep everything straight and figure out what I was supposed to” (Anita, 34, cis woman, lesbian, uterine). The presence of a same-gender partner could also be a way of disclosing sexuality, if they were acknowledged and introduced as such, as Oscar (27 cis man, gay, lymphoma) explained, “the minute my partner came to things like to appointments with me, it was pretty clear.” Participants said that having people around them during cancer who could provide “emotional”, “moral” (Ellen, 36, cis woman, lesbian, uterine) and “practical” support was “reaffirming”, as Carter (20, cis man, gay, leukemia) commented:

Everybody that I was involved with, my parents, friends, family, everybody sort of rallied around me. [It was] really, really reaffirming in myself that I was... It increased my self-worth, that... you know, people were invested in me as a person.

Participants explained that it took time and required intentional action to build supportive communities around themselves, as Ellen (36, cis woman, lesbian, uterine) commented: “It's taken me several years to curate a community for myself, but I've done it. The people who I have now are community-oriented, social justice minded, intersectionality-thinking people who really, really give a shit”.

#### LGBTQI AYAs navigate limited support

A minority of participants were vulnerable to lack of support and isolation due to the combination of their younger age and still developing social networks, and the impacts of minority stressors including anti-LGBTQI prejudice from family and school friends. Nine participants (*n* = 9, 11.5%) reported that they had “no support people during their cancer experience”. Alex (35, non-binary, gay, testicular) said, “at the time, I didn't have anybody I could have with me [at appointments]” because “first of all, I didn't get along with my parents” and because “I also do not have any really close friends that I felt I could actually probably rely on.” Some participants explained that they had “no connection with immediate or extended biological family due to…well, my family are quite awful. And they don’t like queers” (survey, 38, cis female, queer, medical intervention). Others said they lacked family support as “family can be quite toxic” including one survey participant, (survey, 30, cis woman, queer, lung), who said they had been “told by my parent I have cancer because I’m gay”. For a number of participants, family were “around” during cancer treatment, but support was conditional on concealing their sexual and/or gender identities:

My parents don't know I am queer and my partner is a trans woman. So when they're around, my partner will present as male. She only just came out as trans two years ago. We were planning on coming out much sooner but then cancer happened. I don't want to rock the boat right now because I don't think my parents are going to be very accepting. I don't want to have to deal with that on top of cancer. (Brianna, 26, cis woman, queer and asexual, lymphoma)

Many participants also lacked a supportive network of friends during cancer. Some participants said their “friends are too busy” (survey, 33, non-binary, gay, prostate) to provide the support they needed. Others said they experienced anti-LGBTQI prejudice when they came out to their school friends and hadn’t yet developed new social networks before being diagnosed with cancer. For example, Aaron (32, cis man, gay, colorectal) explained that “none of my school friends were accepting of me being gay” and that when he moved from his country hometown to live in a capital city:

Everyone I met was through the clubbing scene, so I didn't really have any friends that I could rely on. If I wasn’t gay, then I would probably have a lot of friends from high school. So, I’d have that sort of resource network there, that sort of natural resource.

Anita (34, cis woman, lesbian, uterine) commented that unlike older people with cancer who often “had a strong network and all of that stuff… I didn't necessarily have all of that so it's a very different experience.”

Sixty (67.4%) participants agreed that they “felt supported by other LGBTQI people during cancer”. However, participants reported that, it could be “hard” to call on support from their LGBTQI chosen family and friends as these people were often experiencing their own life stressors.

With multiple and complex traumas in our communities (there is evidence to support this, I’m not just talking about people I know), higher suicide rates, mental health, homelessness, lower employment... asking for help from partner/s and chosen family can be hard. We are stretched thin and exhausted by this world and its cruelty. Trauma lives on in our collective lives, in our personal histories and the histories we share across multiple identities and communities (Survey, 37, queer femme, unknown primary cancer).

Many participants also lacked an intimate partner; only half of our participants (*n* = 47, 49.5%) were in a relationship, including 39 (41.1%) with one partner and 8 (8.4%) with multiple partners. For some in relationships, cancer “really put a strain on our relationship and led to the end of our relationship” (Oscar, 27, cis man, gay, lymphoma). Dylan (32, non-binary, gay, leukaemia) explained that for them, the difficulties of cancer were exacerbated by lack of support from family which placed strain on their intimate relationships: “I don't really have the support of my family. So, I ended up putting all of that on a partner, and that became too much”. Participants said there was a need for improved support for their carers, particularly LGBTQI partners and other chosen family and friends, as Jessie (37, non-binary and genderfluid, queer, BRCA mutation) commented:

For me, particularly, the LGBTQIA [LGBTQI Aboriginal] community, we need support for our partners as much as we need support for ourselves and we need support for our like non-sexual, non-romantic partners. My primary partner is Aboriginal and there's nothing in that space for Aboriginal Queer women. There should be something out there that overlaps in some way and there just isn't…. It's all really white, and white Australian. My partners have not always been white, and they felt actively excluded from all of the materials I brought home for their sexuality, gender and race.

Brianna (26, cis woman, queer and asexual, lymphoma) explained that support to maintain the capacity of her carer was crucial as “it's like I'm teetering on the edge. If anyone that is supporting me right now decides that they want to stop supporting me, I'm going to be in a lot of trouble.”

#### Finding cancer peer support networks is difficult for LGBTQI AYAs

Participants described difficulties building or finding cancer peer support networks and reported that they often felt “isolated” (Howard, 33, trans man, queer, uterine) as the intersections between being young, LGBTQI and having cancer made it difficult to meet other people with similar experiences. Dylan (32, non-binary, gay, leukemia) said “having a super rare cancer, at this age, and then being queer on top, is just not really a thing”. Cara (29, cis woman, gay, melanoma) commented, “even in the waiting room. The majority of people are over 70. So, I feel like it makes it really difficult to find other people who you can talk to.” Luca (33, non-binary, queer, bladder) explained, “when it comes to cancer, you need somebody that gets it – someone who understands cancer, ideally the type of cancer you have, and potentially comes from the same background or demographics as you”. However, she said she was “yet to meet someone that is LGBTQ (sic) with the same type of cancer in the same age as me” despite having “scoured the globe for young females with a similar cancer and presentation.” Brianna (26, cis woman, queer and asexual, lymphoma) also commented:

It's difficult to find young people with cancer. While it's not exactly difficult to find queer people on the Internet, it's difficult to find people who have the same interests or experiences as you do.

Many participants also said they struggled to find cancer support groups where they fit in, including Alex (35, non-binary, gay, testicular) who said, “there were no support groups for men in my situation—gay and single. There were support groups for the family man, but that didn’t interest me at all because that's not who I am.” Whilst some participants said the “internet sort of filled that gap”, others found online support groups to either be hostile or unhelpful. Flynn (34, non-binary, queer, uterine) was “kicked out” of an online support group for young people with their cancer type “for asking them to please not be racist transphobes”. They said, “I had one of the only sources of information completely ripped out of my hands because they wanted to be racist more than they wanted to help people”. Howard (33, trans man, queer, uterine) said that “having autism […] plus being trans plus cancer” made it hard “really hard finding specific help” because “not many people in the population are Trans, not many people have sensory issues. And so, it was nearly impossible to find support”.

In combination, the accounts in these three subthemes demonstrate the importance of social support for LGBTQI AYAs with cancer and the precarious nature of social support experienced by some individuals.

## Discussion

This study is the most substantive study to date to examine experiences of cancer and cancer care among LGBTQI AYAs across a range of ages, sexual and gender identities and tumor types. Qualitative interview and open-ended survey data are integrated with closed ended survey data to provide rich insights into the experiences and support needs of LGBTQI AYAs with cancer. Participants’ accounts evidenced how cancer could disrupt critical processes of accepting, exploring and articulating LGBTQI identities and communities, leaving those diagnosed to play “catch up” with identity work after completing treatment. Anti-LGBTQI prejudice meant that many participants lacked support from family and/or friends, feared discrimination and avoided disclosing their sexual and/or gender identities in cancer care. Cis-heteronormativity contributed to the invisibility of LGBTQI AYAs in cancer care, information and support. The intersection of this population’s age and LGBTQI status resulted in multiple forms of marginalization in a healthcare system already ill-equipped to provide tailored holistic care to either AYAs [44] or LGBTQI communities [45]. Further, the unique issues and concerns reported by participants help to explain why our study found higher rates of distress among LGBTQI AYAs relative to LGBTQI older adults [13], as well as why previous studies evidenced poorer psychological wellbeing amongst LGBTQI AYAs with cancer, relative to cisgender heterosexual counterparts [11, 12].

A common theme across AYA cancer experiences is the element of biographical disruption: the disruption of one's self-concept and identity, expected life course, and the very structures of daily life by a chronic or serious illness [42, 46–49]. LGBTQI AYAs may experience additional unique challenges as they navigate still-developing LGBTQI identities, relationships and community connections in tandem with the impacts of illness, treatment and survivorship. This paper is the first to describe how cancer can interrupt identity exploration and establishment for LGBTQI AYAs, with participants describing having to relegate these processes to “the backburner” for some, while still struggling to accept and articulate these aspects of themselves. Interrupting this identity work may be distressing for AYAs, particularly if this involves ongoing internalized prejudice or delaying identity-affirming processes [50, 51]. Additionally, if AYAs have not disclosed that they are LGBTQI to HCPs, friends or family, they may lack appropriate support to navigate the impacts of cancer and treatment on LGBTQI identities, relationships and sexuality, and may continue to receive cis- and heteronormative cancer care and information [16, 17]. As reported in the general AYA cancer literature [25], participants also noted that physical and sexual changes impacted their relationships and sexual activity post-cancer treatment; this presented a further challenge to LGBTQI identities and connection to community, as has been previously reported for older LGBTQI people with cancer [22, 52, 53]. Affected AYAs may benefit from tailored support that addresses physical and sexual concerns in the context of LGBTQI identities and relationships and helps to connect them to accessible and supportive queer communities.

Cis-heteronormative assumptions within cancer care have contributed to the invisibilization and exclusion of LGBTQI people from cancer care, resources, and information [21, 54]. The present findings extend this research in highlighting the unique challenges faced by LGBTQI AYAs with cancer in navigating these difficulties. Like older LGBTQI people with cancer [21, 52], AYAs in this study reported challenges disclosing their sexual and gender identities to medical professionals, including fear of hostility and discrimination. These fears were linked to previous mistreatment. Being a young LGBTQI person adds a layer of complexity in that AYAs may lack experience and confidence to disclose to HCPs, or correct cis-normative or hetero-normative assumptions. Disclosure and integration of one’s sexual and gender identity within one’s cancer care are associated with better health outcomes [55]. For AYAs in this study, avoidance of disclosure was associated with feelings of inauthenticity, a lack of holistic care, and inadequately tailored care for their needs. However, AYAs whose HCPs were aware they were LGBTQI faced the very real prospect of discrimination in their cancer care; almost half of all participants, and almost three-quarters of trans participants, reported experiencing discrimination as part of their care. This places LGBTQI AYAs with a difficult dilemma: with each new HCP encountered, they must assess whether it is safe and feasible to disclose or allow HCP assumptions to persist and potentially receive care and information inappropriate for their needs.

These difficulties may exacerbate known issues for the general AYA population around inadequate information provision and exclusion from treatment decision-making, associated with HCP assumptions about their emotional state and maturity level [56], dissatisfaction with care, and subsequent poorer health outcomes. This is particularly true for AYAs treated in the paediatric sector [57], with medical professionals reluctant to discuss sensitive topics such as sexuality and fertility with younger AYAs, despite the importance of these topics to this age group [58]. Both AYAs and LGBTQI people with cancer are more likely to report unmet needs around sexuality and fertility [55, 57], and the intersection of these discrepancies in care may render LGBTQI AYAs particularly vulnerable to receiving inadequate cancer care and information – particularly as LGBTQI-specific information is also lacking [55]. Given that many AYAs depend on their HCPs as their primary source of information [59], it is crucial for HCPs to be able to facilitate disclosure of LGBTQI status and respond appropriately, discuss sexuality, gender identity and fertility, and make LGBTQI-specific information available to patients.

Our findings confirmed and extended previous research that social support is important to wellbeing throughout the cancer continuum [11], including for AYAs and LGBTQI people with cancer [15]. However, LGBTQI AYAs with cancer are vulnerable to isolation due to difficulties accessing care and support from their family of origin and friends. For non-LGBTQ AYAs with cancer, the family of origin are often a primary source of support during cancer [15]. However, due to anti-LGBTQ hostility, LGBTQ AYAs may be rejected by their family of origin when they “come out”, removing this main source of support, with additional financial consequences due to absence of economic support and secure accommodation. Alternately, LGBTQ AYAs may need to conceal their sexual and gender identities and relationships when receiving support from their family of origin, adding to minority stress. For older LGBTQI patients, cancer caregivers are typically intimate partners and chosen family and friends, particularly those who are LGBTQI themselves [29]. Support from other LGBTQI people offers group solidarity, affirms LGBTQI identities and relationships [60] and can help patients navigate cis-heteronormativity and discrimination in cancer care. However, younger LGBTQI people may not have partners or strong LGBTQI community connections, while similarly aged peers may lack the resources or understanding to provide support; in the broader Out with Cancer study, AYAs were less likely than older adults to report support from LGBTQI community [13, 29] and may therefore be unable to compensate for an unsupportive family of origin.

Like previous reports [61] cancer patients often want to connect with and receive support from other patients who have similar experiences and challenges. Whilst for some of our participants online communities facilitated these connections, in general, LGBTQ AYAs lacked peer support during cancer. Previously reported issues connecting small, geographically dispersed populations like AYAs with cancer may be exacerbated by cisnormativity, heteronormativity, and other types of invisibility or discrimination in peer support spaces [19, 21]. This meant that participants had fewer opportunities to connect with and learn from other LGBTQI AYAs, limiting their cancer-specific social support [62]. These findings highlight the potential vulnerabilities of LGBTQI AYAs to lack of support and isolation during cancer, especially for those with multiple marginalized identities such as LGBTQI AYAs from diverse cultural backgrounds, LGBTQI AYA Indigenous Australians, LGBTQI AYAs living with disability, and those living in rural regions. There is a need for targeted cancer peer support groups and networks for LGBTQI AYAs, and these support systems must also be made available to LGBTQI caregivers.

### Strengths and limitations

The mixed-methods approach, combined with the exploration of psychosocial experiences across multiple domains, helps explain the previously evidenced psychosocial vulnerability of LGBTQI AYAs [13]. There was considerable diversity in the genders, sexualities and cancer stages and types represented amongst participants, allowing a more complete understanding of how a broad sample of LGBTQI AYAs may experience cancer and cancer care. Further research is needed to examine the ways in which LGBTQI AYAs navigate cancer and cancer care at different stages, including at first diagnosis and recurrent diagnosis, and whether this differs across cancer types. Participants were predominantly white people from Australia and other Western countries; further research is needed to explore the experiences of LGBTQI AYAs from other ethnic groups and in other countries, and the ways in which different care systems may impact on distress levels. The sample was also largely comprised of older AYAs with high health literacy, including healthcare workers and student healthcare professionals who may have more experience and agency than younger AYAs in cancer care. Our sample also contained a low number of intersex people, and all of the intersex people identified as gender or sexuality diverse (LGBT). Further research is needed to explore the experiences of younger LGBTQI AYAs and particularly those with an intersex variation, many of whom identify as heterosexual [63]. Additionally, future studies should explore the experiences of LGBTQI AYAs with advanced or terminal diagnoses, who may have particularly acute needs around balancing an authentic sense of self with retaining support systems at the end of life.

An important consideration when working with LGBTQI communities is the variability in age ranges used to define adolescence and young adulthood. The definition used by this study (16–39 years) is broad, and may include individuals at different stages of understanding, embracing and expressing their identities, from those who are who have not realised or come to accept their LGBTQ identity, to those who are secure in their identities, communities and relationships. However, it is also important to note that older LGBTQI people may also be undergoing processes often associated with adolescence and young adulthood, such as the exploration of emerging identities, relationships and sexuality – particularly if societal or internalised prejudices have prevented them from realising or expressing LGBTQI identities previously. That is, while the issues highlighted in this paper are considered characteristic of AYAs, it is important to be aware that they are not exclusive to this age group.

## Conclusions and implications

LGBTQI AYAs with cancer experience psychosocial vulnerabilities related to their identity development, experiences of care, and social support networks. These factors likely contribute to their previously evidenced elevated risk of distress, relative to both non-LBGTQI AYAs [11, 12] and LGBTQI older adults [13]. Our work identifies these three areas as key domains where LGBTQI AYAs affected by cancer may require additional, tailored supportive care, including targeted information resources, LGBTQI and/or AYA specific cancer support groups, or partnerships and referrals to LGBTQI community organisations. Additionally, it is evident that HCPs and cancer services have much work to do in ensuring LGBTQI AYAs receive affirming and appropriate care across paediatric and adult clinical settings. It is important that service providers recognise the broader sociocultural context that shapes this population’s experiences of cancer and cancer care, particularly regarding the invisibilisation and marginalization LGBTQI communities have faced both within and outside of healthcare systems. They must move beyond assuming all patients are cisgender, heterosexual and do not have intersex variations unless otherwise stated, work to signal inclusivity and facilitate disclosure, and be able to respond appropriately with tailored information and care, which is inclusive of LGBTQI partners, chosen family, and support systems [19, 21]. To this end, it is essential to extend research and knowledge translation efforts in this area, ensuring that the insights gained in this work are used to effect meaningful change in how tailored care is provided to this population.

## Data Availability

The datasets used and/or analysed during the current study are available from the corresponding author on reasonable request.

## Abbreviations

AYA: Adolescents and young adults
BRCA: BRCA BReast CAncer gene
HCP: Health care professionals
LGBTQI: Lesbian, gay, bisexual, transgender, queer and/or intersex
LGBQ: Same gender attracted—Lesbian, gay, bisexual, queer
Trans: Transgender
Cis: Cisgender
